# Functional Characterization of an Arylsulfonamide-Based Small-Molecule Inhibitor of the NLRP3 Inflammasome

**DOI:** 10.1021/acschemneuro.4c00512

**Published:** 2024-09-19

**Authors:** Savannah Biby, Prasenjit Mondal, Yiming Xu, Ashley Gomm, Baljit Kaur, Jannatun N. Namme, Changning Wang, Rudolph E. Tanzi, Shijun Zhang, Can Zhang

**Affiliations:** † Department of Medicinal Chemistry, Virginia Commonwealth University, Richmond, Virginia 23298, United States; ‡ Genetics and Aging Research Unit, McCance Center for Brain Health, MassGeneral Institute for Neurodegenerative Disease, Department of Neurology, Massachusetts General Hospital, Harvard Medical School, Charlestown, Massachusetts 02129, United States; § Athinoula A. Martinos Center for Biomedical Imaging, Department of Radiology, Massachusetts General Hospital, Harvard Medical School, Charlestown, Massachusetts 02129, United States

**Keywords:** neuroinflammation, NOD-like receptor family pyrin domain containing 3 (NLRP3), inhibitor, binding interaction, microglia, phagocytosis

## Abstract

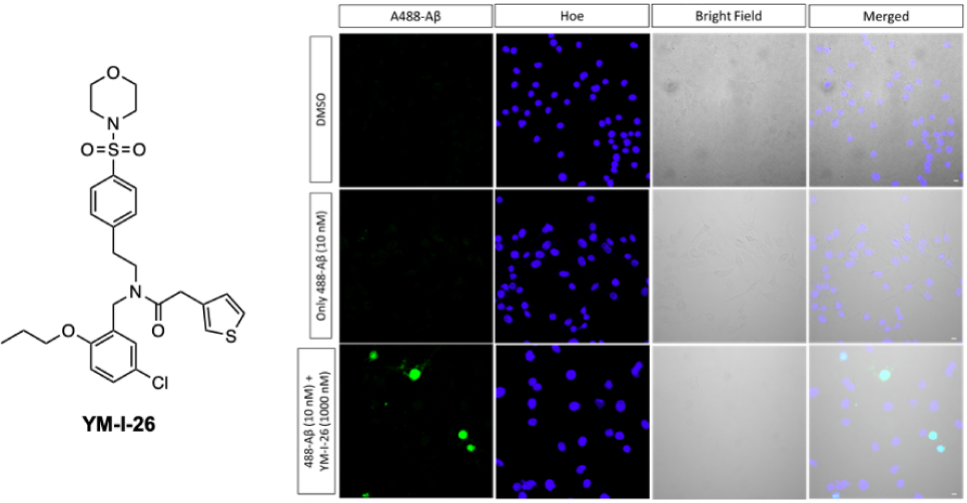

Considerable evidence indicates that the NOD-like receptor family pyrin domain-containing 3 (NLRP3) inflammasome plays key roles in human pathophysiology, suggesting it as a potential drug target. Currently, studies have yet to develop compounds that are promising therapeutics in the clinic by targeting the NLRP3 inflammasome. Herein, we aim to further biologically characterize a previously identified small-molecule inhibitor of the NLRP3 inflammasome from our group, YM-I-26, to confirm its functional activities. We showed that YM-I-26 is highly selective toward the NLRP3 inflammasome and binds to NLRP3 directly. A systemic analysis revealed YM-I-26 with inflammation-related and immunomodulatory activities by the Eurofins BioMAP Diversity PLUS panel. In addition, studies using the mouse microglia BV2 cell model demonstrated that YM-I-26 is not cytotoxic, improved the phagocytotic functions of BV2 cells toward beta-amyloid, and suppressed the production of cytokines of IL-1β and IL-10 upon the activation of the NLRP3 inflammasome. Collectively, our studies support the functional activities of YM-I-26 as a NLRP3 inhibitor in physiologically relevant cell models, and warrant future studies of YM-I-26 and its analogs to advance the drug development as potential therapeutics.

## Introduction

The NOD-like receptor family pyrin domain-containing 3 (NLRP3) inflammasome is a cytosolic multimeric protein complex and an essential component of innate immunity that tightly regulates inflammatory responses. Activation of the NLRP3 inflammasome leads to the production of pro-inflammatory cytokines, particularly interleukin (IL)-1β and IL-18, and promotes inflammatory responses.^1−4^ There is considerable evidence implicating the critical roles of dysregulated NLRP3 inflammasome in the pathogenesis of various disorders, e.g., Alzheimer’s disease (AD) and pain.^5−10^ As a result, the NLRP3 inflammasome has been deemed a viable drug target for a variety of human diseases,^11−15^ and NLRP3 inhibitors (NLRP3is) have been actively pursued to achieve disease interventions.^16−27^

**Figure 1:**
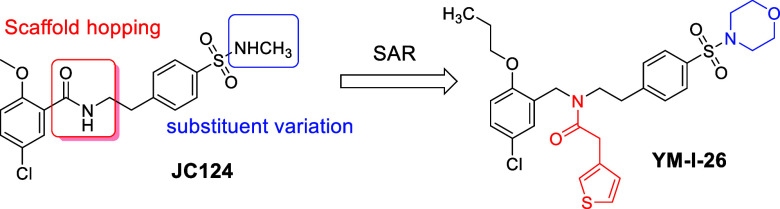
Chemical structure of YM-I-26.

Efforts from our group have led to the discovery of NLRP3is based on a sulfonamide-containing scaffold, and studies in preclinical animal models demonstrated their *in vivo* efficacy to ameliorate disease pathology and improve functions by engaging the NLRP3 inflammasome.^28−37^ Among our previously reported NLRP3is, YM-I-26 (Figure 1), was selected for further characterization to understand its mechanism of action and functional activities given its unique structural features and demonstrated potency. To this end, biophysical, molecular modeling, and physiologically relevant cell models were employed. The results from the studies revealed that YM-I-26 exhibits activities to modulate inflammation- and immune-related functions via direct interactions with NLRP3, thus hinting its clinical potential and encouraging further investigations.

## Results and Discussions

**Figure 2:**
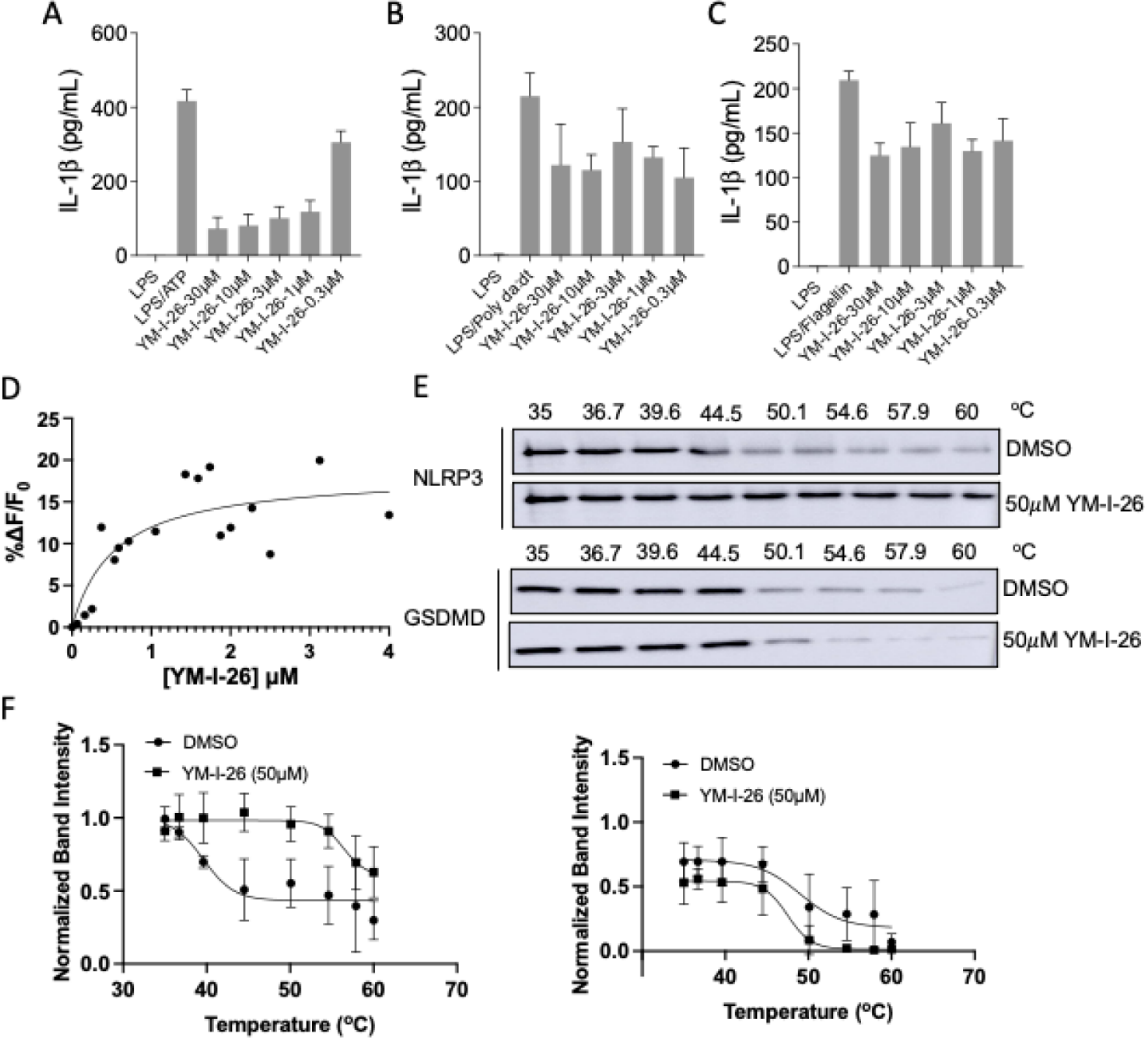
YM-I-26 selectively inhibits the NLRP3 inflammasome and binds to recombinant NLRP3. (A) J774A.1 cells were primed with LPS (1 μg/mL) for 4.5 h and then treated with various concentrations of YM-I-26, followed by stimulation with ATP (5 mM) for 30 min. IL-1β in the culture media was measured by ELISA. (B) J774A.1 cells were treated with LPS (1 μg/mL) and various concentrations of YM-I-26 for 1 h, followed by stimulation with (poly(dA:dT)) (4 μg/mL) for 4 h or (C) Flagellin (1 μg/mL) for 6 h. The supernatants were collected and levels of IL-1β were measured by ELISA. (D) Binding affinity of YM-I-26 with recombinant mouse NLRP3 tested by fluorescence spectroscopy (FS). (E, F) Recombinant mouse NLRP3 or GSDMD was incubated with or without 50 μM YM-I-26 and heated at indicated temperatures followed by Western blot analysis. Images is representative of three independent tests (E). Normalized band intensities determined from ImageJ analysis (F).

Our previous studies established that YM-I-26 inhibited the release of IL-1β upon LPS/ATP stimulation in murine macrophage J774A.1 cells and no cytotoxicity was observed for this compound at up to 10 μM.^37^ Dose–response studies in J774A.1 cells upon the activation of inflammasomes confirmed its potent inhibition on the NLRP3 inflammasome with an IC_50_ of 0.65 ± 0.14 μM (Figure 2A) but minimal inhibition on the AIM2 (Figure 2B) or NLRP4 (Figure 2C) inflammasome, consistent with our previous report.^37^ Binding studies using a fluorescence spectroscopy method demonstrated the direct interaction of YM-I-26 with the full length recombinant NLRP3 protein with a *K*_*d*_ of 560.2 ± 30.3 nM (Figure 2D). Thermal shift studies of recombinant NLRP3 protein in the absence or presence of YM-I-26 (Figure 2E,F) revealed a positive shift in the melting point of NLRP3 from 41.14 ± 1.64 to 57.59 ± 1.97 °C. We also examined its interaction with gasdermin D (GSDMD), an immediate downstream substrate of the NLRP3 inflammasome that is responsible for the release of cytokines via pore formation,^38,39^ using the thermal shift experiment under the same experimental settings. As shown in Figure 2E,F, minimal impact was observed on GSDMD in the presence of YM-I-26. This further supports that the observed inhibition on IL-1β release by YM-I-26 is via its specific binding interactions with NLRP3.

**Figure 3:**
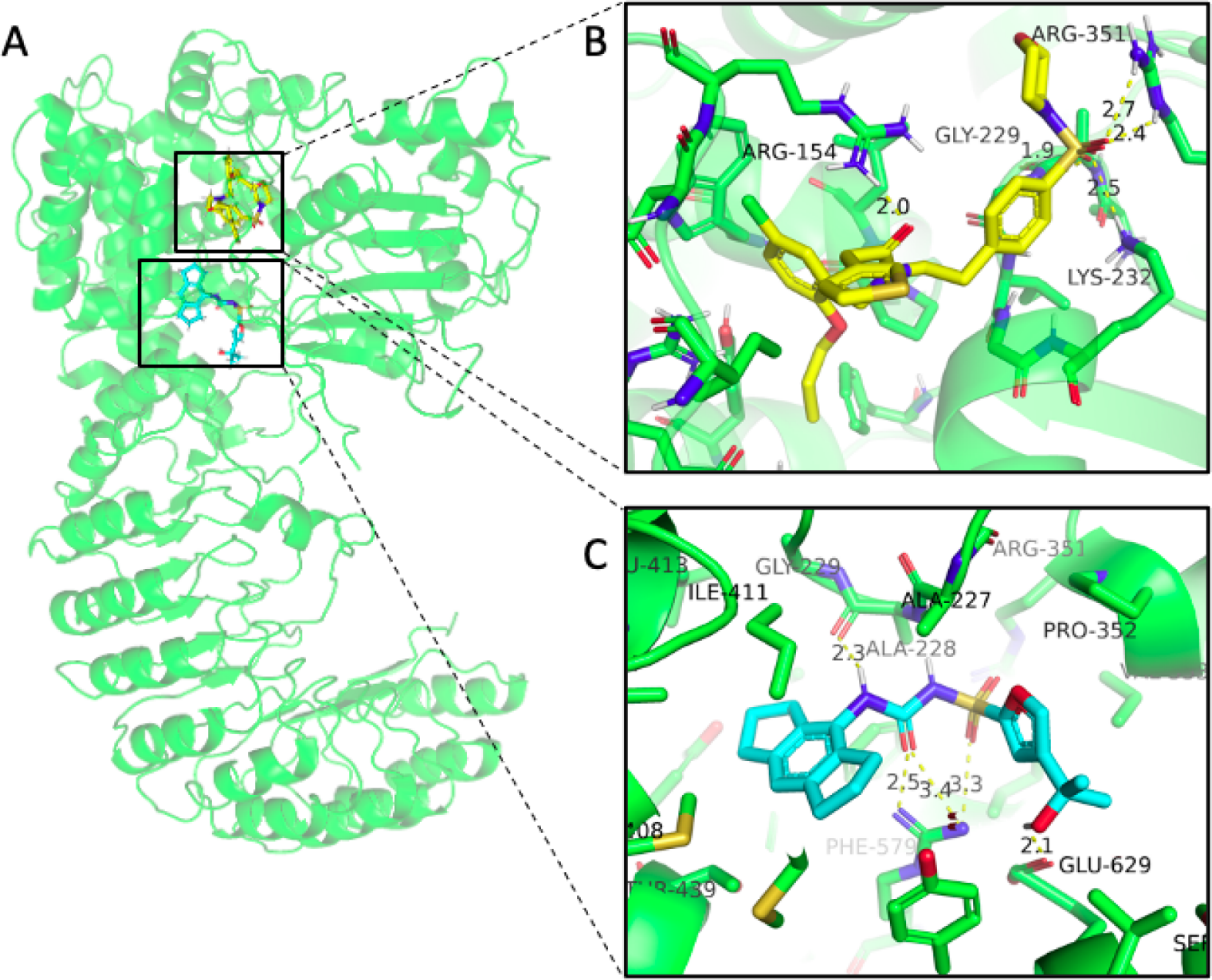
YM-I-26 preferentially binds to the ATP site of NLRP3. (A) Superimposed images of YM-I-26 and MCC950 binding to their respective binding sites. (B) Predicted binding model of YM-I-26 with NLRP3 (PDB: 8EJ4). (C) Cryo-EM structure of MCC950 binding to NLRP3 (PDB: 7PZC). Dashed lines represent possible hydrogen bonding interactions between compound and residues.

To better understand its binding mode within the NLRP3 protein, molecular modeling studies were performed. As shown in Figure 3A, YM-I-26 fits favorably to the ATP binding site compared to the MCC950 binding site (PLP score of 93.3 and 85.2, respectively). As shown in Figure 3B, the 5-chloro-2-propoxybenzyl moiety and the 4-(phenylsulfonyl)morpholine moiety of YM-I-26 form hydrophobic interactions within the ATP binding pocket. In addition, multiple H-bond interactions were observed between the sulfonyl moiety and GLY-229 (1.9 Å), ARG-351 (2.4 and 2.7 Å), and LYS-232 (2.5 Å), as well as between the amide moiety and ARG-154 (2.0 Å) (Figure 3B). Overall, YM-I-26 adopted a T-shape structure under this binding mode in which the thiophene moiety of YM-I-26 is oriented to a solvent-exposed region and blocks the access of ATP to the binding cavity. In comparison, MCC950 favors an allosteric site instead of the ATP binding site, with its tricyclic hexahydro-s-indacene moiety and the furan moiety of MCC950 forming hydrophobic interactions, while its sulfonylurea moiety forms H-bond interactions with ALA228 (2.3 Å) and PHE579 (2.5 and 3.3 Å) (Figure 3C). An additional H-bond interaction was also observed between the 2-hydroxypropan with GLU629 (2.1 Å). Collectively, the docking results suggest a distinct binding mode of YM-I-26 compared to MCC950 with NLRP3.

**Figure 4:**
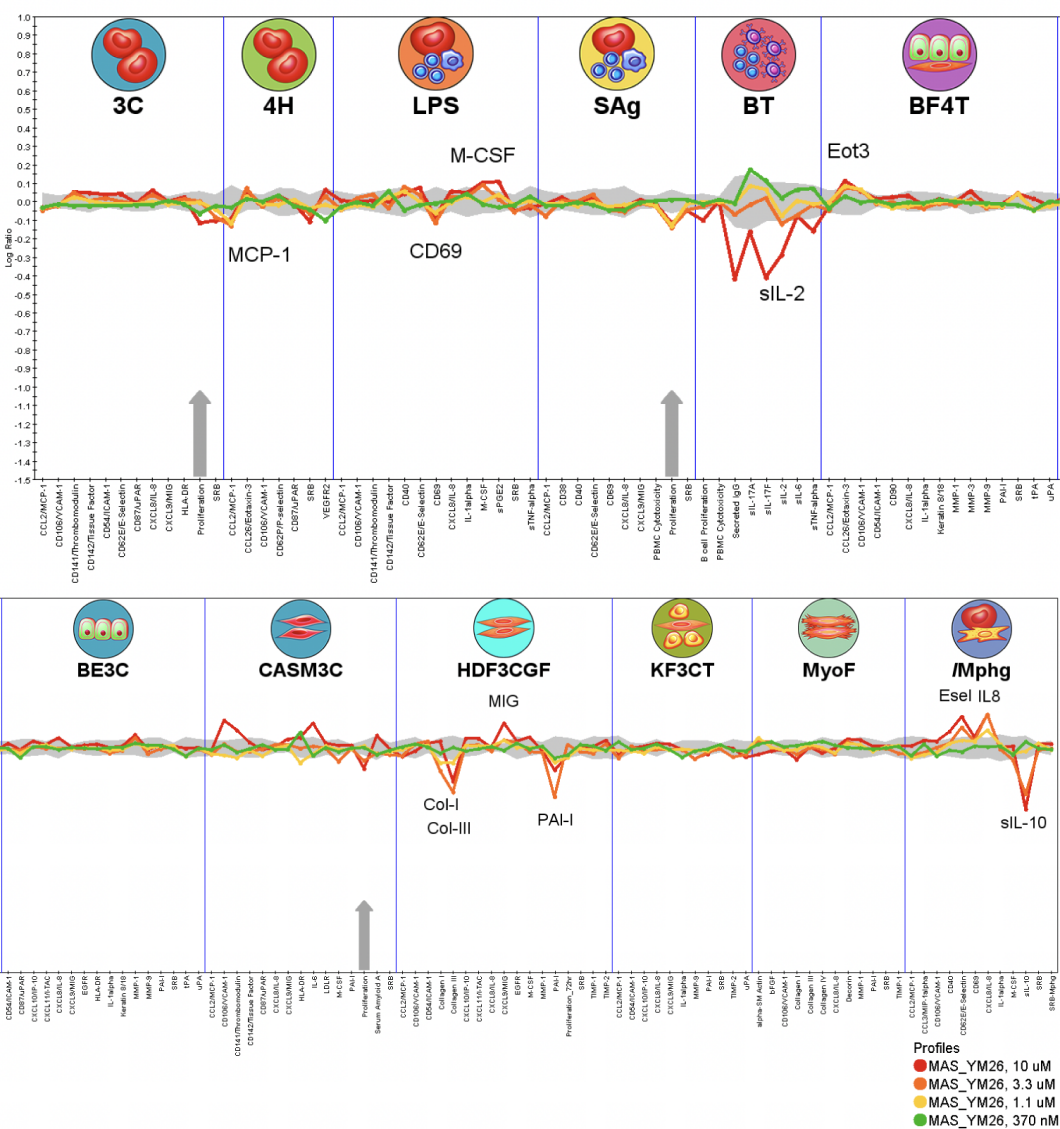
Functional characterization of YM-I-26 by the Eurofins BioMAP profiling in the Diversity PLUS Panel. The *X*-axis lists the quantitative protein-based biomarker readouts measured in each system. The *Y*-axis represents a log-transformed ratio of the biomarker readouts for the drug-treated sample (*n* = 1) over vehicle controls (*n* ≥ 6). The gray region around the *Y*-axis represents the 95% significance envelope generated from historical vehicle controls. Biomarker activities are annotated when two or more consecutive concentrations change in the same direction relative to vehicle controls, are outside of the significance envelope, and have at least one concentration with an effect size >20% (|log10 ratio| > 0.1). Biomarker key activities are described as modulated if these activities increase in some systems but decrease in others. Cytotoxicity is indicated on the profile plot by a thin black arrow above the *X*-axis, and antiproliferative effects are indicated by a thick gray arrow. Cytotoxicity and antiproliferative arrows only require one concentration to meet the indicated threshold for profile annotation.

After confirming that YM-I-26 is a direct NLRP3i with unique binding mode to NLRP3, we next employed the BioMAP Diversity PLUS panel to analyze its functional activities. This panel contains 12 human primary cell-based systems (Table S1)^40,41^ and is designed to evaluate the therapeutic and biological relevance of testing compounds and to validate their safety, specific drug effects and disease outcomes. As illustrated in Figure 4, YM-I-26 was tested at 0.37, 1.1, 3.3, and 10 μM concentrations. Notably, no cytotoxicity was observed at all tested concentrations, while antiproliferative activities in venular endothelial cell and coronary artery smooth muscle cells at 10 μM were noted. The analysis also revealed that YM-I-26 exhibited modulatory effects on inflammation-related and tissue remodeling activities by increasing activities on systems LPS (M-CSF), BF4T (Eot3)/Mphg (Esel, IL8), and HDF3CGF (MIG) while decreasing activities in 4H (MCP1), HDF3CGF (Col-I, Col-III, PAI-I), /Mphg (sIL-10), LPS (CD69, M-CSF), and BT (sIL-2) (Table 1). The observed anti-inflammatory activities of YM-I-26 may be via its inhibition on the release of proinflammatory cytokines as demonstrated in Figure 2A by our studies. Furthermore, the changes in Col-I, Col-III, and PAI-I in the HDF3CGF system by YM-I-26 suggest that the tissue remodeling-related activity of YM-I-26 could be due to its modulation of Th1-mediated inflammation, which has been shown to have a significant impact on this fibrotic system.^41−47^

**Table 1:** Twelve Annotated Readouts Upon Treatment with YM-I-26

Biological and Disease Relevance Category	Decreased Activity	Increased Activity
Inflammation-related activities	MCP-1	Eot3, MIG, Esel, IL-8
Immunomodulatory activities	sIL-10, sIL-2, CD69	
Tissue remodeling activities	Col-I, Col-III, PAI-I	M-CSF

**Figure 5:**
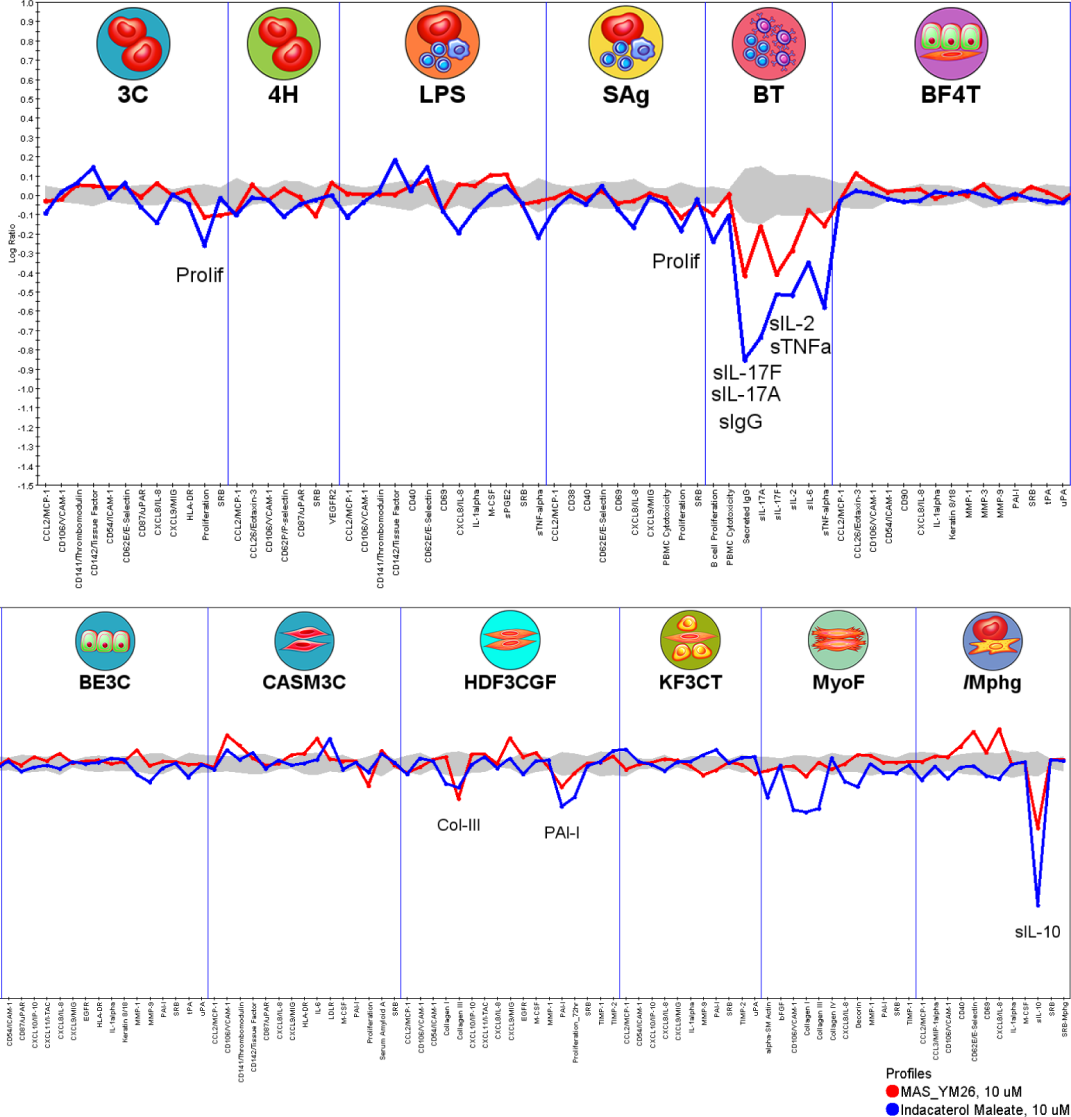
Functional comparison of YM-I-26 with indacaterol maleate for mechanisms of action in Eurofins BioMAP profiling from the Diversity PLUS Panel.

Further mechanistic similarity analysis by an unsupervised search of the BioMAP Reference Database of >4,500 agents yielded a top match between YM-I-26 and indacaterol maleate (Figure 5), a β2 adrenergic receptor agonist. The Pearson’s score for this match is 0.72, above the preset threshold of *r* ≥ 0.7 (Table S2). Indeed, the literature has shown that β2 adrenergic receptor agonism is one of the key drivers in controlling inflammation through the regulation of IL-10, and has been suggested to have therapeutic potential in neurodegenerative disorders.^48^

**Figure 6:**
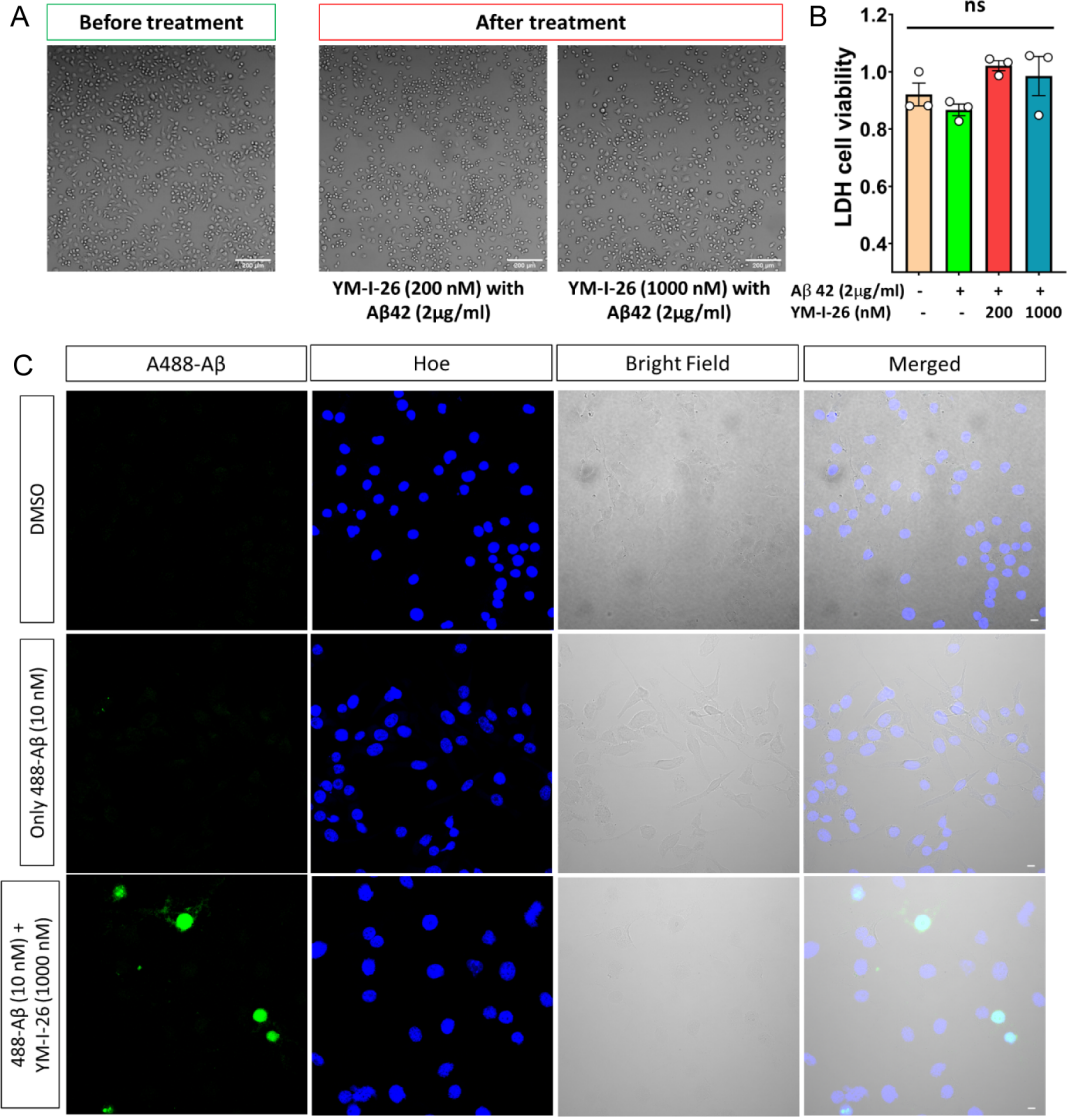
YM-I-26 shows no cytotoxicity and increases the phagocytotic activities in BV2 cells. (A) Confocal bright field (10× objective) images of BV2 cell before and after treatment with YM-I-26. (B) LDH levels were measured from the cell culture media under indicated conditions. No significant difference was observed upon YM-I-26 treatment in BV2 cell line. (C) Confocal microscopy of BV2 cell treated with Alexa488-Aβ shows upregulation of phagocytosis in the presence of YM-I-26. Scale bars correspond to 10 μm.

**Figure 7:**
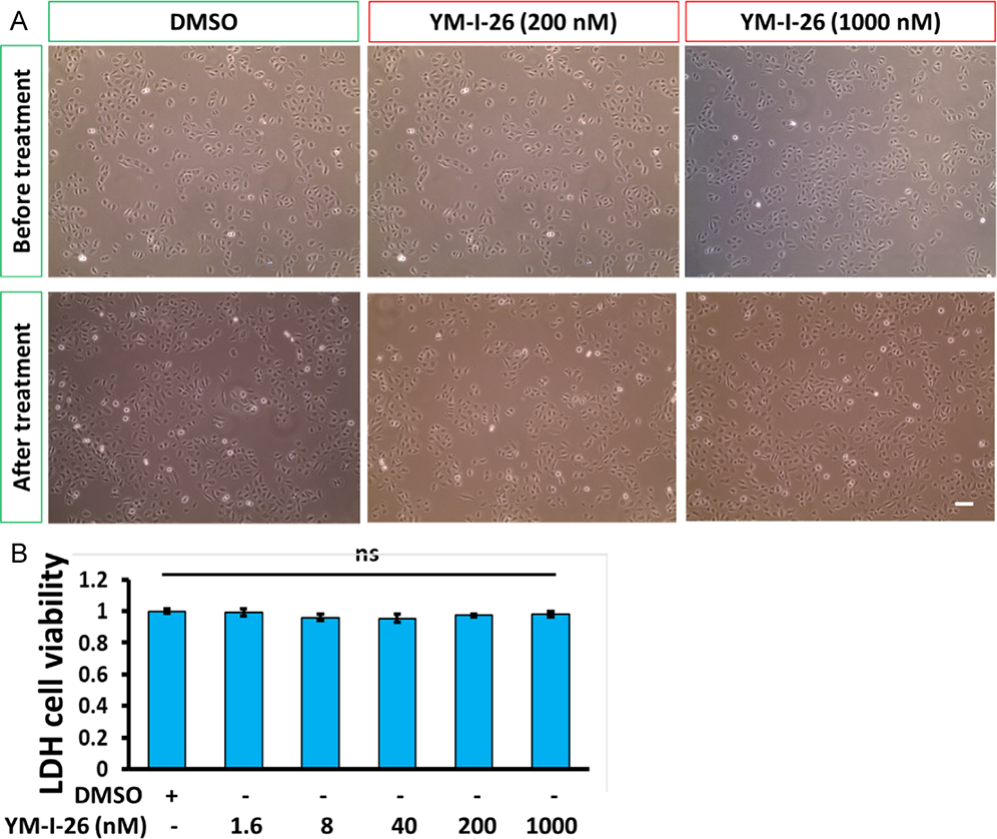
YM-I-26 shows no cytotoxicity in H4-APP751 cells. (A) Stereo microscopic images of H4-APP751 cell before and after treatment with YM-I-26. (B) LDH assay shows no significant difference upon treatment with YM-I-26 in H4-APP751 cell line. Scale bar corresponds to 100 μm. ns: nonsignificant.

Given the observed inflammation- and immunomodulation-related activities by YM-I-26 from the Eurofins BioMAP Diversity PLUS Panel analysis, we then analyzed the effects of YM-I-26 on NLRP3-related innate immune responses using mouse microglia BV2 cells focusing on both safety and two primary microglia-related functions, phagocytosis and regulation of proinflammatory cytokine. First, we treated BV2 cells with YM-I-26 and analyzed the potential cytotoxicity of this compound. In addition, BV2 cells were exposed to beta-amyloid 42 (Aβ42), the toxic species that has been shown to play essential roles in cognitive impairment in AD,^49−52^ followed by treatment with different concentrations of YM-I-26 to measure the change in phagocytosis. Stereo microscopic images taken before and after YM-I-26 treatment indicated no detrimental effect of this compound (Figure 6A). LDH toxicity assay confirmed this notion by showing no significant changes in the presence of YM-I-26 (Figure 6B). Confocal microscopic images indicated higher Alexa488-Aβ positive signal in the presence of YM-I-26 compared to only Alexa488-Aβ42-treated cells (Figure 6C). Thus, YM-I-26 led to upregulation of Aβ phagocytosis, suggesting microglia-mediated functional benefits in clearance. Further studies in human neuroglioma H4-APP751 cells validated the general safety of YM-I-26 as evidenced by the morphological analysis (Figure 7A) and LDH assay (Figure 7B).

**Figure 8:**
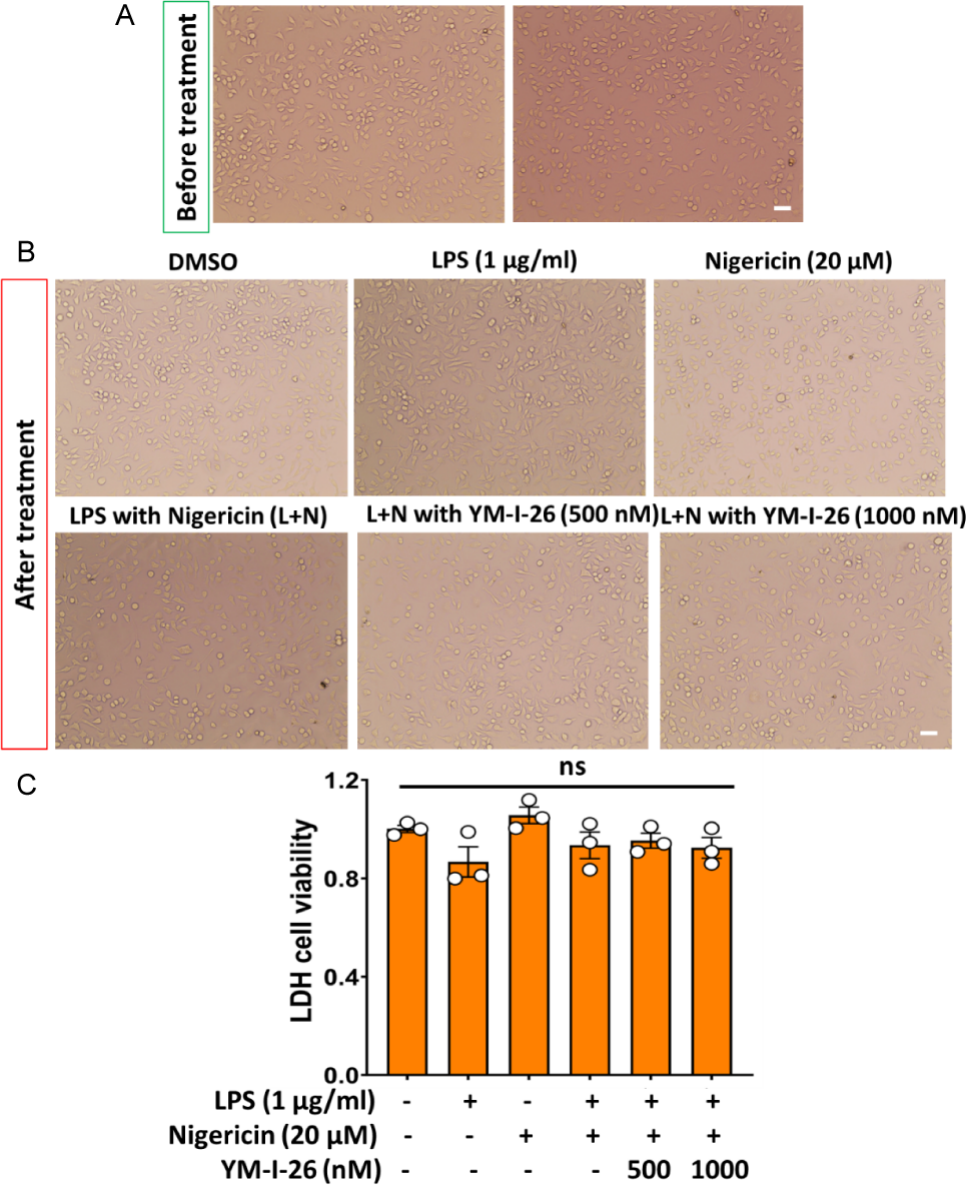
Stereo microscopic images of BV2 cell before (A) and after (B) treatment with LPS (1 μg/mL), Nigericin (20 μM), and LPS + Nigericin (L+N) with different concentrations of YM-I-26. (C) LDH assay shows no significant difference indicates no cytotoxicity of YM-I-26 in BV2 cell line even in NLRP3 inflammatory cell condition. ns: nonsignificant.

**Figure 9:**
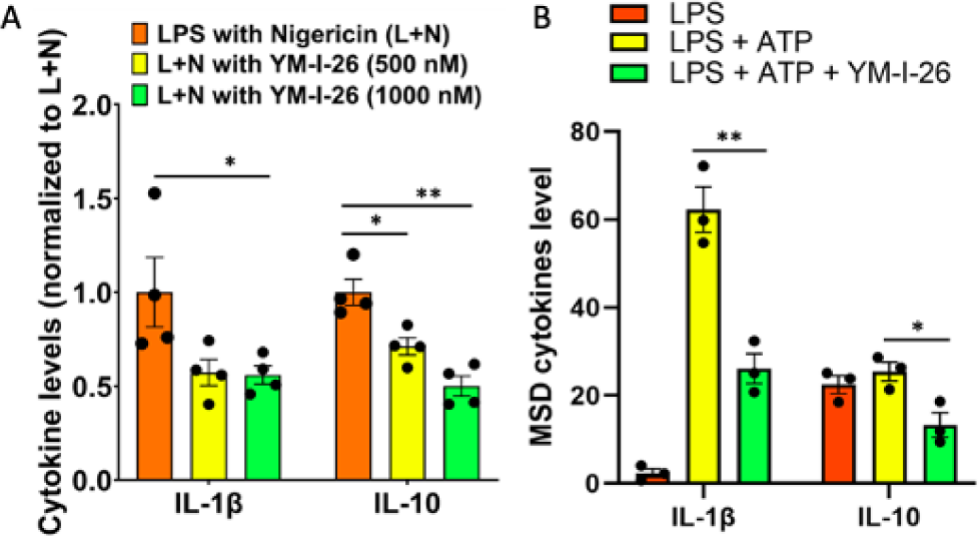
YM-I-26 treatment suppressed the production of IL-1β and IL-10 in microglia BV2 cells. MSD cytokines assay was performed to measure the cytokines protein levels from media collected from YM-I-26-treated BV2 cells activated by LPS/Nigericin (A) or LPS/ATP (B). The results demonstrated significant reduction of IL-1β and IL-10 levels. Results re-expressed as mean ± SEM (*n* = 3); Student’s *t-*test (*p < 0.05; **p < 0.001).

Next, we evaluated the effects of YM-I-26 in BV2 cells under an environment in which the NLRP3 inflammasome is activated. Morphologically, no impact was observed by YM-I-26 treatment upon the activation of the NLRP3 inflammasome by LPS (1 μg/mL) and nigericin (20 μM) (Figure 8A,B). LDH assay of the cell media also indicated no significant toxicity of YM-I-26 (Figure 8C) under this experimental setting, consistent with the results from aforementioned cytotoxicity studies. Further MSD-proinflammatory cytokines analysis using the cell media found that YM-I-26 treatment led to significant reductions of IL-1β (Figure 9A), supporting the roles of YM-I-26 as an NLRP3i. Interestingly, YM-I-26 treatment was also associated with reduced levels of IL-10 (Figure 9A), consistent with the findings from the analysis by the Eurofins BioMAP Diversity PLUS Panel (Figure 3). Upon activation of the NLRP3 inflammasome in BV2 cells by LPS (1 μg/mL) and ATP (5 mM), YM-I-26 treatment consistently resulted in a significant reduction in IL-1β levels (Figure 9B). Notably, YM-I-26 treatment also led to a significant reduction in the IL-10 levels under this experimental setting (Figure 9B). This further confirms the potent activity of YM-I-26 as an NLRP3i.

## Summary

The biological discovery of NLRP3 inflammasome in association with the pathogenesis of various human disorders has driven drug development to reduce neuroinflammation by engaging NLRP3 with small molecule NLRP3is. Our recent efforts embarked on development of sulfonamide-based NLRP3is and exploring their potential application in AD and other neurodegenerative disorders.^28−36^ Here, we further profiled one of our previously reported NLRP3is,^37^ YM-I-26, through comprehensive characterizations using biophysical assays, molecular modeling, and various cellular models. The studies indicated that YM-I-26 is a potent and selective NLRP3i that directly binds to the ATP binding site of NLRP3, different from the known inhibitor MCC950. The results from the Eurofins BioMAP Diversity PLUS panel analysis revealed inflammation-related and immunomodulatory activities upon YM-I-26 treatment. Notably, the systems impacted by YM-I-26 treatment recapitulate Th1 inflammatory responses driven by immune cells (LPS and/Mphg systems). Changes were also annotated by the analysis of inflammation related markers, such as MCP-1, Esel, IL-8, Eot3, and MIG. Taken together, this provides strong evidence to support the roles of NLRP3 inhibition by this compound and potentially other NLRP3is in the observed activities. Biological characterization in the murine microglia BV2 model confirmed that YM-I-26 is not cytotoxic, consistent with the results from the BioMAP Diversity PLUS panel study. Importantly, YM-I-26 treatment in BV2 cells led to decreased level of IL-1β and IL-10 under two different NLRP3 activation mechanisms. Again, this confirms the inhibitory activity of this compound on the NLRP3 inflammasome instead of upstream targets. The suppression of IL-10 also echoes the results from the BioMAP Diversity PLUS panel analysis. Notably, YM-I-26 treatment increased phagocytotic activities of BV2 cells to Aβ42, an essential microglia function to clear Aβ and other pathological debris. Therefore, the combination of inflammatory suppression and increased phagocytosis by YM-I-26 strongly suggests its potential clinical benefits. Collectively, the results from the comprehensive and systematic characterization of YM-I-26 by employing murine microglia and human primary cell-based systems encourage further investigation and development of YM-I-26 and its analogs as potential therapeutic agents with clinical benefits.

## Materials and Methods

### Chemical Synthesis

YM-I-26 was synthesized as previously described,^37^ and stock solutions were prepared in DMSO.

### Microglial BV2 Cell-Based Experiments

BV2 cells were cultured following previous published methods^53^ and grew in DMEM media containing 10% heat-inactivated fetal bovine serum, 2 mM l-Glutamine, and 1% penicillin/streptomycin (Life Technologies). For treatment, BV2 cells were first incubated with DMSO (vehicle) or lipopolysaccharide (LPS) (1 μg/mL) for 4 h, then incubated with YM-I-26 in the presence of inflammasome activators, either nigericin (20 μM) or ATP (5 mM) for 30 min. Subsequently, media was collected and assayed for LDH to assess cell viability; cells were extensively washed with PBS and were lysed in MPER lysis buffer (MPER++) supplemented with EDTA-free protease inhibitors (Roche), Halt phosphatase inhibitor cocktail (Thermo Fisher Scientific). Lysates were centrifuged^53^ (12,000 rpm at 4 °C for 15 min) and supernatants were collected for further analysis.

### MSD Analysis for Inflammatory Cytokines

We performed previously reported MSD immunoassays that were based on multiarray technology in combination with electrochemiluminescence to identify multiple proteins in a single sample. The methods were based on previous reports.^54,55^

### Western Blotting (WB) Analysis

WB analysis was performed following previously reported procedure.^54^ Briefly, cell lysates were collected and then protein concentrations were measured using the Pierce BCA protein kit (Pierce 23225). Equal amount proteins were applied to the WB analysis. Proteins were separated by electrophoresis using the 4–12% Bis-Tris SDS-PAGE Gel System (Invitrogen, Thermo Fisher Scientific), followed by membrane transfer to PVDF membranes (Invitrogen, Thermo Fisher scientific). Next, the membranes were blocked for 30 min with the Superblock (Thermo Fisher Scientific) and then incubated overnight with primary antibodies. The membranes were washed with TBST and incubated with secondary antibodies (1:10,000 dilution) at room temperature for 2 h. Then, membranes were incubated with Supersignal West Femto (Life Technologies) and the images for immunoreactivity were visualized by the LICOR Odyssey Fc. Quantifications of proteins of interest were performed by ImageJ software.

### Inhibition of the NLRP3 Inflammasome

J774A.1 cells were plated into 96-well plates (1 × 10^5^ cells/well) in growth medium overnight. Cells were primed with *Escherichia coli* 0111:B4 LPS (Sigma-Aldrich) (final concentration: 1 μg/mL) for 4.5 h. YM-I-26 was then added and incubated for 30 min. After incubation, ATP (5 mM) was added to activate the NLRP3 inflammasome for 30 min. Supernatants were then collected and IL-1β level was measured with a mouse IL-1β ELISA kit (DuoSet ELISA, R&D Systems) following the manufacturer’s instructions.

### Inhibition of the NLRC4 and AIM2 Inflammasomes

J774A.1 cells were plated into 96-well plates (1 × 10^5^ cells/well) in growth medium overnight. After removal of the growth media, cells were treated with LPS (1 μg/mL) and various concentrations of YM-I-26 for 1 h. Flagellin (1 μg/mL) (Enzo Life Sciences, Farmingdale, NY) or polydeoxyadenylic-deoxy-thymidylic acid sodium salt (poly(dA:dT)) (4 μg/mL) (InvivoGen, San Diego, CA) in DMEM was added to activate the NLRC4 or AIM2 inflammasome, respectively. Flagellin cell-transfection was accomplished utilizing the Polplus transfection kit (PULSin, New York, NY). For AIM2 activation, cells were incubated with poly(dA:dT) delivered by Lipofectamine 2000 Transfection Reagent (Thermo Fisher Scientific) for 4 h. The supernatants were collected and IL-1β level was measured with a mouse IL-1β ELISA kit following the manufacturer’s instructions.

### Recombinant Protein Production and Purification

The recombinant NLRP3 and GSDMD proteins were expressed and purified as previously described.^56,57^ Briefly, Expi293F cells were transfected with pcDNA3-N-Flag-NLRP3 (Addgene plasmid # 75127)^2^ by ExpiFectamine 293 Transfection Kit (Thermo Fisher Scientific). After 48 h, cells were harvested and suspended in lysis buffer (50 mM HEPES, 150 mM NaCl, 1 mM TCEP, 10 mM MgCl_2_, 1x protease inhibitors, 10% glycerol, and 1% DDM, pH 7.5). Cell lysates were centrifuged at 50,000 × *g* at 4 °C for 1 h. Supernatant was incubated with Mouse IgG agarose beads (Sigma-Aldrich) at 4 °C, followed by incubation with M2 Anti-FLAG beads (Sigma-Aldrich) at 4 °C for 2–3 h. The beads were then washed with lysis buffer and eluted with elution buffer (50 mM HEPES, 500 mM NaCl, 10% glycerol, 0.1% DDM, and 100 μg/mL FLAG peptide (MedChemExpress, Thermo Fisher Scientific), pH 7.5). The eluted fractions were concentrated. For the production of GSDMD, BL21(DE3) cells were transformed with pDB-His-MBP-mGSDMD (Addgene plasmid # 123365)^58^ and induced with 0.5 mM IPTG. Cells were then collected and lysed in lysis buffer (25 mM Tris-HCl, 150 mM NaCl, 20 mM imidazole, 5 mM 2-ME, 1x protease inhibitors, pH 8.0). Cell lysates were centrifuged at 25,000 × *g* at 4 °C for 1 h and supernatant was purified using HisPur Ni-NTA resin (Thermo Fisher Scientific). The eluted fractions were pooled and concentrated.

### Fluorescence Spectroscopy

The fluorescence spectroscopy assay was conducted as described previously.^56^ The Cary Eclipse Fluorescence Spectrophotometer (Agilent Technologies) was used to measure the intrinsic fluorescent intensity (ex: 280 nm; em: 340 nm) of the recombinant mouse NLRP3 protein (500 nM). The protein was added into a fluorescence micro cell cuvette (SKU: 6610021600) and measured for initial fluorescence, followed by compound titration. The percent change in fluorescence was calculated for each point and the titration was carried out until a plateau in the curve was achieved. Data was fitted using a hyperbolic nonlinear curve regression in the Prism software to determine the *K*_d_ value.

### Thermal Shift Assay

The thermal shift assay was conducted as previously described.^59^ Briefly, recombinant mNLRP3 or mGSDMD protein was incubated with YM-I-26 (50 μM) or DMSO on ice for 1 h. The mixture was equally divided into eight PCR tubes and heated at 35, 36.7, 39.6, 44.5, 50.1, 54.6, 57.9, and 60 °C simultaneously for 10 min for NLRP3 or 3 min for GSDMD. The samples were then centrifuged for 20 min at 18,000 × *g* and 4 °C, followed by SDS-PAGE gel electrophoresis and Western Blot analysis to determine the NLRP3 or GSDMD levels in each sample.

### Computational Modeling

The docking study was conducted as previously reported.^60^ Briefly, the NLRP3 crystal structure (PDB ID: 8EJ4)^61^ and cryo-EM structure of NLRP3 (PDB: 7PZC)^62^ were used. Protein structure was prepared in GOLD5.6, and this includes removal of water molecules, extraction of native AGS ligand, and addition of polar hydrogen atoms. The structures of the docked ligands were prepared in SYBYL-X 2.1.1 software. After adding all hydrogen atoms and energy minimization, ligands were docked. The top 10 binding poses for each protein–ligand complex were analyzed and interactions were visualized by PyMOL 2.3.4 software.

### Eurofins BioMAP Diversity PLUS Panel Analysis

Detailed protocols for the BioMAP Diversity PLUS panel of 12 human primary cell/coculture systems and related analysis, including benchmark, similarity and mechanism HeatMAP analysis, have previously been published.^40,41,63−66^

### Lactate Dehydrogenase (LDH) Assay

Following the manufacturer’s guidelines, CytoTox-ONE kit (Promega) was utilized to analyze cell viability. Briefly, cell culture media (50 μL, collected after treatment with YM-I-26) was mixed with substrate in 1:1 dilution, placed in a round-bottom clear 96-well plate and incubated at 37 °C for 30 min. Then, the plate was read using a spectrophotometer at an excitation wavelength of 560 nm to measure the different signal intensity. Fluorometric signal intensities of control groups were compared with the treated groups to assess the cytotoxicity of treated compounds.

### Stereomicroscopic Cell Images

Stereomicroscopic images were captured before and after the treatment of YM-I-26 on BV2 and H4-APP751 cells to visualize the detrimental effect of YM-I-26 on these cells. Cytotoxicity of compound was assessed based on cellular morphology of BV2 and H4-APP751cells.

### Immunocytochemistry (ICC) Study to Visualize the Phagocytosis of YM-I-26-Treated BV2 Cells

ICC was performed to visualize the effect of YM-I-26 on microglial phagocytosis. BV2 cells were seeded at ∼100,000 cells/well and grown overnight in a LabTek 8 well chamber. Carefully, the cells were washed twice with PBS and treated with DMSO, and Alexa-488-Aβ (10 nM) with or without YM-I-26 (1000 nM). After 3 h, the media was discarded, each well was washed carefully with PBS twice and fixed with 4% paraformaldehyde (PFA) for 20 min at room temperature. Then, each well was washed twice again with PBS and incubated with 10 nM of Hoechst solution for 10 min at room temperature, washed with PBS twice, and finally mounted with Prolong Gold antifade reagent, dried at room temperature, and covered with a coverslip. Microscopic images were captured afterward.

### Data Analysis

ImageJ analysis software was used to process and analyze the microscopic and immunoblotting images. All the results for different experiments in this work were shown as mean ± SEM. Statistical significance of the results were performed using Student’s two tailed *t-*test in GraphPad PRISM. Values were considered as significant when *p* values are less than 0.05.
