# Supplementary material for: Functional Characterization of an Arylsulfonamide-Based Small-Molecule Inhibitor of the NLRP3 Inflammasome
Source: ACS Chem Neurosci. 2024 Sep 19;15(19):3576–86. doi: 10.1021/acschemneuro.4c00512 (PMC11450741; doi:10.1021/acschemneuro.4c00512)
Supplement: Supplementary file 1 — cn4c00512_si_001.pdf [file cn4c00512_si_001.pdf]

Supplementary Information

Functional characterization of an arylsulfonamide-based small molecule inhibitor of the NLRP3 inflammasome

Savannah Biby[a], Prasenjit Mondal[b], Yiming Xu[a], Ashley Gomm[b], Baljit Kaur[a], Jannatun N. Namme[a], Changning Wang[c], Rudolph E. Tanzi[b], Shijun Zhang[a, *], Can Zhang[b,*]

[a]*Department of Medicinal Chemistry, Virginia Commonwealth University, Richmond, Virginia 23298, United States*

[b]*Genetics and Aging Research Unit, McCance Center for Brain Health, MassGeneral Institute for Neurodegenerative Disease, Department of Neurology, Massachusetts General Hospital, Harvard Medical School, Charlestown, Massachusetts, 02129, United States*

[c]*Athinoula A. Martinos Center for Biomedical Imaging, Department of Radiology, Massachusetts General Hospital, Harvard Medical School, Charlestown, Massachusetts 02129, United States*

** Co-corresponding authors*

Corresponding Authors
Shijun Zhang — *Department of Medicinal Chemistry, Virginia Commonwealth University, Richmond, Virginia 23298, United States;*
Email: szhang2@vcu.edu
Can Zhang — *Genetics and Aging Research Unit, McCance Center for Brain Health, MassGeneral Institute for Neurodegenerative Disease, Department of Neurology, Massachusetts General Hospital, Harvard Medical School, 114 16th Street, Charlestown, Massachusetts, 02129, United States.*
Email: zhang.can@mgh.harvard.edu

Table S1. The 12 systems in the BioMAP Diversity PLUS panel, including a list of the cell types, disease context and list of biomarker readouts optimized for each system.

System Name	Icon	Human Cell Types	Stimulation	Disease/Tissue Relevance	Biomarker Readouts	System Description
3C	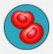	Venular endothelial cells	TNFα, IL-1β, IFNγ	Cardiovascular Disease, Chronic Inflammation	CCL2/MCP-1, CD106/VCAM-1, CD141/Thrombomodulin, CD142/Tissue Factor, CD54/ICAM-1, CD62E/E-Selectin, CD87/uPAR, CXCL8/IL-8, CXCL9/MIG, HLA-DR, Proliferation, SRB	The 3C system models Th1 type vascular inflammation and is an anti-angiogenic environment that promotes monocyte and T cell adhesion and recruitment. The 3C system is relevant for chronic inflammatory diseases, vascular inflammation and restenosis.
4H	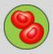	Venular endothelial cells	IL-4, Histamine	Autoimmunity, Allergy, Asthma	CCL26/Eotaxin-3, CCL2/MCP-1, CD106/VCAM-1, CD62P/P-Selectin, CD87/uPAR, SRB, VEGFR2	The 4H system models Th2 type vascular inflammation and is a pro-angiogenic environment that promotes mast cell, basophil, eosinophil, T and B cell recruitment. The 4H system is relevant for diseases where Th2-type inflammatory conditions play a role such as allergy, asthma, and ulcerative colitis.
LPS	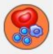	Venular endothelial cells, Peripheral blood mononuclear cells	TLR4 ligand	Chronic Inflammation, Cardiovascular Disease	CCL2/MCP-1, CD106/VCAM-1, CD141/Thrombomodulin, CD142/Tissue Factor, CD40, CD62E/E-Selectin, CD69, CXCL8/IL-8, IL-1 alpha, M-CSF, sPGE2, SRB, sTNF-alpha	The LPS system models Th1 type chronic inflammation and monocyte activation responses. The LPS system is relevant to chronic inflammatory conditions where monocytes play a key role including atherosclerosis, restenosis, rheumatoid arthritis, and metabolic diseases.
SAg	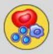	Venular endothelial cells, Peripheral blood mononuclear cells	TCR ligands (1X)	Chronic Inflammation, Autoimmune Disease	CCL2/MCP-1, CD38, CD40, CD62E/E-Selectin, CD69, CXCL8/IL-8, CXCL9/MIG, PBMC Cytotoxicity, Proliferation, SRB	The SAg system models Th1 type chronic inflammation and T cell effector responses related to T cell proliferation and activation in the context of the vascular endothelium. Modeling chronic inflammation of the Th1 type and T cell effector responses, the SAg system is relevant for T-cell driven inflammatory conditions including organ transplantation, rheumatoid arthritis, psoriasis, Crohn's disease and hematological oncology.
BT	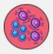	Peripheral blood mononuclear cells, B cells	α-IgM, TCR ligands (0.001X, sub-mitogenic levels)	Asthma, Oncology, Autoimmunity, Allergy	B cell Proliferation, PBMC Cytotoxicity, Secreted IgG, sIL-17A, sIL-17F, sIL-2, sIL-6, sTNF-alpha	The BT system models the T cell dependent B cell proliferation, activation and class switching that occurs in the germinal centers of secondary lymphoid organs. The BT system is relevant for indications in which B cell activation and antibody production have been implicated including systemic lupus erythematosus (SLE), hematological oncology, autoimmune indications, asthma and allergy.
BF4T	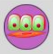	Bronchial epithelial cells, Dermal fibroblasts	IL-4, TNFα	Fibrosis, Lung Inflammation, Asthma, Allergy	CCL26/Eotaxin-3, CCL2/MCP-1, CD106/VCAM-1, CD54/ICAM-1, CD90, CXCL8/IL-8, IL-1 alpha, Keratin 8/18, MMP-1, MMP-3, MMP-9, PAI-I, SRB, tPA, uPA	The BF4T system models Th2 type lung inflammation and is an environment that promotes the recruitment of eosinophils, mast cells and basophils as well as effector memory T cells. The BF4T system is relevant for allergy and asthma, pulmonary fibrosis, as well as COPD exacerbations.
BE3C	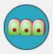	Bronchial epithelial cells	IL-1β, IFNγ, TNFα	COPD, Lung Inflammation	CD54/ICAM-1, CD87/uPAR, CXCL10/IP-10, CXCL11/I-TAC, CXCL8/IL-8, CXCL9/MIG, EGFR, HLA-DR, IL-1 alpha, Keratin 8/18, MMP-1, MMP-9, PAI-I, SRB, tPA, uPA	The BE3C system models Th1 type lung inflammation and is an environment that promotes monocyte and T cell adhesion and recruitment. The BE3C system is relevant for sarcoidosis and pulmonary responses to respiratory infections.
CASM3C	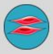	Coronary artery smooth muscle cells	IL-1β, TNFα, IFNγ	Cardiovascular Inflammation, Restenosis	CCL2/MCP-1, CD106/VCAM-1, CD141/Thrombomodulin, CD142/Tissue Factor, CD87/uPAR, CXCL8/IL-8, CXCL9/MIG, HLA-DR, IL-6, LDLR, M-CSF, PAI-I, Proliferation, Serum Amyloid A, SRB	The CASM3C system models Th1 type vascular inflammation and is an environment that promotes monocyte and T cell recruitment. The CASM3C system is relevant for chronic inflammatory diseases, vascular inflammation and restenosis.
HDF3CGF	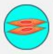	Dermal fibroblasts	IFNγ, TNFα, IL-1β, EGF, bFGF, PDGF-BB	Fibrosis, Chronic Inflammation	CCL2/MCP-1, CD106/VCAM-1, CD54/ICAM-1, Collagen I, Collagen III, CXCL10/IP-10, CXCL11/I-TAC, CXCL8/IL-8, CXCL9/MIG, EGFR, M-CSF, MMP-1, PAI-I, Proliferation_72hr, SRB, TIMP-1, TIMP-2	The HDF3CGF system models wound healing and matrix/tissue remodeling in the context of Th1-type inflammation. The HDF3CGF system is relevant for various diseases including fibrosis, rheumatoid arthritis, psoriasis as well as stromal biology in tumors.
KF3CT	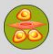	Keratinocytes, Dermal fibroblasts	IL-1β, IFNγ, TGFβ, TNFα	Dermatitis, Psoriasis	CCL2/MCP-1, CD54/ICAM-1, CXCL10/IP-10, CXCL8/IL-8, CXCL9/MIG, IL-1 alpha, MMP-9, PAI-I, SRB, TIMP-2, uPA	The KF3CT system models model Th1 type cutaneous inflammation and is an environment that promotes monocyte and T cell adhesion and recruitment. The KF3CT system is relevant for cutaneous responses to tissue damage caused by mechanical, chemical, or infectious agents as well as certain states of psoriasis and dermatitis.
MyoF	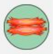	Lung fibroblasts	TGFβ, TNFα	Wound Healing, Matrix Remodeling, Fibrosis, Chronic Inflammation	alpha-SM Actin, bFGF, CD106/VCAM-1, Collagen I, Collagen III, Collagen IV, CXCL8/IL-8, Decorin, MMP-1, PAI-I, SRB, TIMP-1	The MyoF system models general myofibroblast differentiation and tissue remodeling relevant for multiple fibrotic diseases. Biomarker readouts capture impacts on translationally relevant matrix remodeling, tissue repair and inflammation related responses in fibrotic tissue.
/Mphg	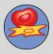	Macrophages, Venular endothelial cells	TLR2 ligand	Chronic Inflammation, Restenosis, Cardiovascular Disease	CCL2/MCP-1, CCL3/MIP-1 alpha, CD106/VCAM-1, CD40, CD62E/E-Selectin, CD69, CXCL8/IL-8, IL-1 alpha, M-CSF, sIL-10, SRB, SRB-Mphg	The /Mphg system models Th1 type chronic inflammation and macrophage activation responses. The /Mphg system is relevant to inflammatory conditions where monocytes play a key role including atherosclerosis, restenosis, rheumatoid arthritis, and other chronic inflammatory conditions.

Table S2. Similarity analysis of YM-I-26 using the BioMAP profiling reference database.

MAS_YM26	Database Match	BioMAP Z-Standard	Pearson's Score	# of Common Readouts	Mechanism Class
10 µM	Indacaterol Maleate, 10 µM	10.920	0.720	148	Long-acting β2 Adrenergic Receptor Agonist
	Temsirolimus, 1.1 µM	10.669	0.709	148	mTOR Inhibitor
	BEZ235, 37 nM	10.553	0.705	148	PI3K Inhibitor
3.3 µM	Bindarit, 200 µM	7.360	0.545	148	CCL2/7/8 Inhibitor
	GSK2801, 10 µM	7.118	0.531	148	BAZ2 Bromodomain Inhibitor
	DuP 128, 3.3 µM	6.514	0.510	137	ACAT Inhibitor
1.1 µM	Bindarit, 200 µM	5.290	0.413	148	CCL2/7/8 Inhibitor
	DuP 128, 3.3 µM	5.127	0.416	137	ACAT Inhibitor
	AKT Inhibitor VIII, 1.1 µM	5.039	0.445	114	Akt Inhibitor
370 nM	Bromosporine, 14 nM	6.389	0.486	148	Pan Bromo Domain Inhibitor
	Humalog, 4.1 mU/ml	5.730	0.443	148	Insulin Receptor Agonist
	Emtricitabine (Emtriva), 3.3 µM	5.723	0.465	132	Reverse transcriptase inhibitor

Top matches for YM-I-26 were listed including the molecules and their doses in the profiling analysis. A table of the top three similarity matches from an unsupervised search of the BioMAP Reference Database of >4,500 agents for each concentration of test agent. The similarity between agents is determined using a combinatorial approach that accounts for the characteristics of BioMAP profiles by filtering (Tanimotometric) and ranking (BioMAP Z-Standard) the Pearson's correlation coefficient between two profiles. Profiles are identified as having mechanistically relevant similarity if the Pearson's correlation coefficient is ≥ 0.7. The Pearson's correlation coefficient between profiles that is above our determined threshold (r ≥ 0.7) indicates the compounds share mechanistically relevant similarity.
